# Risk scores’ performance and their impact on operative decision-making in left-sided endocarditis: a cohort study

**DOI:** 10.1007/s10096-022-04516-2

**Published:** 2022-11-08

**Authors:** A. Fernández-Cisneros, M. Hernández-Meneses, J. Llopis, E. Sandoval, D. Pereda, J. Alcocer, C. Barriuso, M. Castellá, J. Ambrosioni, J. M. Pericàs, B. Vidal, C. Falces, C. Ibáñez, J. Perdomo, I. Rovira, C. García-de-la-María, A. Moreno, M. Almela, A. Perisinotti, A. Dahl, P. Castro, J. M. Miró, E. Quintana, Equip d’Endocarditis de l’Hospital Clínic de Barcelona

**Affiliations:** 1 Cardiovascular Surgery Department, Hospital Clínic - IDIBAPS, University of Barcelona, C/Villarroel 170, 08036 Barcelona, CP Spain; 2 Infectious Diseases Service, Hospital Clínic - IDIBAPS, University of Barcelona, Barcelona, Spain; 3 Department of Genetics, Microbiology and Statistics, University of Barcelona, Barcelona, Spain; 4 Departament de Cirurgia i Especialitats Medicoquirúrgiques, Facultat de Medicina i Ciències de la Salut, Universitat de Barcelona, Barcelona, Spain; 5 Liver Unit, Internal Medicine Department, Vall d’Hebron University Hospital, Vall d’Hebron Institute of Research, CIBERehd, Barcelona, Spain; 6 Cardiology Department, Hospital Clinic-IDIBAPS, University of Barcelona, Barcelona, Spain; 7 Anesthesiology Department, Hospital Clínic-IDIBAPS, University of Barcelona, Barcelona, Spain; 8 Microbiology Department, Hospital Clínic-IDIBAPS, University of Barcelona, Barcelona, Spain; 9 Nuclear Medicine Department, Biomaterials and Nanomedicine (CIBER-BBN), Hospital Clinic-IDIBAPS, University of Barcelona & Biomedical Research Networking Center of Bioengineering, Barcelona, Spain; 10 Internal Medicine Department, Hospital Clínic-IDIBAPS, University of Barcelona, Barcelona, Spain

**Keywords:** Risk scores, Infective endocarditis, Postoperative outcomes, Risk stratification

## Abstract

The accuracy of contemporary risk scores in predicting perioperative mortality in infective endocarditis (IE) remains controversial. The aim is to evaluate the performance of existent mortality risk scores for cardiovascular surgery in IE and the impact on operability at high-risk thresholds. A single-center retrospective review of adult patients diagnosed with acute left-sided IE undergoing surgery from May 2014 to August 2019 (*n* = 142) was done. Individualized risk calculation was obtained according to the available mortality risk scores: EuroScore I and II, PALSUSE, Risk-E, Costa, De Feo-Cotrufo, AEPEI, STS-risk, STS-IE, APORTEI, and ICE-PCS scores. A cross-validation analysis was performed on the score with the best area under the curve (AUC). The 30-day survival was 96.5% (95%CI 91–98%). The score with worse area under the curve (AUC = 0.6) was the STS-IE score, while the higher was for the RISK-E score (AUC = 0.89). The AUC of the majority of risk scores suggested acceptable performance; however, statistically significant differences in expected versus observed mortalities were common. The cross-validation analysis showed that a large number of survivors (> 75%) would not have been operated if arbitrary high-risk threshold estimates had been used to deny surgery. The observed mortality in our cohort is significantly lower than is predicted by contemporary risk scores. Despite the reasonable numeric performance of the analyzed scores, their utility in judging the operability of a given patient remains questionable, as demonstrated in the cross-validation analysis. Future guidelines may advise that denial of surgery should only follow a highly experienced Endocarditis Team evaluation.

**Supplementary information:**

The online version contains supplementary material available at 10.1007/s10096-022-04516-2.

## Introduction

Despite diagnostic and therapeutic improvements, postoperative mortality for infective endocarditis (IE) remains high [1–3]. Cardiovascular surgery (CS) will be indicated in nearly half of IE patients [4, 5]. A timely operation to restore hemodynamic function, to eliminate embolic risk, and/or to control infection contributes to survival [6, 7]. Although indications for surgery might be clear from a structural or infective standpoint, in daily clinical practice the decision to offer CS is often challenging. Nearly a quarter of patients with surgical indication do not receive CS [1, 8]. This reality may be a consequence of perceived futility or denial of operative management on the basis of a high-risk score estimate.

The use of risk scores for endocarditis aims at an objective measurement of mortality risk inherent to the disease and may assist into benchmarking healthcare systems. Specific recommendations for clinical practice and individual decision-making based on available risk scores remain an aspect to be further developed in current ESC guidelines for IE [7]. Intending to predict mortality, multiple specific IE risk scores have been developed and adopted [5, 9–15]. Some have also been shown to predict in-hospital mortality even in patients treated without CS [16]. One significant drawback of these scores is their performance in external cohorts [2, 17–20]. At an individual patient level, the indication for CS in the face of extreme operative risk and marginal chances of meaningful survival is controversial.

We aim to explore the performance of multiple operative mortality risk scores in our most contemporary surgical experience of active left-sided IE. In addition, we examine the outcomes at high-risk thresholds to explore the hypothetical loss of life if arbitrary cut-offs for operability had been used.

## Materials and methods

### Patients

All consecutive patients admitted to our institution from 1 May 2014 to 31 August 2019 with diagnosis of definite acute left-sided IE who underwent CS were reviewed. All patients with IE were discussed prospectively and the indications to pursue CS were agreed within the Hospital Clínic Endocarditis Team as stated in other works [21]. Patients’ baseline characteristics and intraoperative, postoperative, and follow-up data were collected from the departmental database, outpatient’s clinic visits, telephone interviews, and referring physicians’ notes.

### Definitions

The diagnosis of definite IE was made according to the modified Duke criteria in all cases [22]. Results were reported following the Guidelines for Reporting Mortality and Morbidity After Cardiac Valve Interventions by Akins [23]. CS was considered urgent when it was performed within the first 7 days following hospital admission and emergent when performed in the first 24 h [7]. Definitions related to the IE pathology process and outcomes follow those already published by the International Collaboration on Endocarditis [14].

### IE-CS mortality risk scores’ performance and QoL assessment

The estimated mortality risk was calculated at the time of surgery for each patient using the most employed scores in CS to predict mortality (STS-risk score and EuroScore I and II) [24–27]. Moreover, we calculated the predicted mortality risk using specific IE-CS risk scores, which include several items known to affect the patients’ outcome as prosthetic valve IE or the existence of a paravalvular complication. These risk scores included PALSUSE [5], Risk-E [11], Costa [9], De Feo-Cotrufo [10], AEPEI [13], the modified STS-IE [28], APORTEI [12], and ICE-PCS scores [14]. Composite morbimortality risk was also assessed by the STS-IE score [28]. Scores were calculated using the same definitions of variables as stated in their original article [5, 9–14, 24–28].

We performed a cross-validation study on the basis of different preoperative mortality risk thresholds. We used arbitrary cut-off points for mortality risk of 45%, 60%, and 70% for each score to assess the impact of operability at these incremental risks. We excluded those scores for which the number of patients with risk over 45% was less than 5 (STS, STS-IE). To better understand the risk prediction, we calculated the predictive positive value (PPV) with its 95% confidence interval (95% CI) for EuroScore II, STS-risk, Risk-E, and ICE-PCS scores. We selected these scores due to their performance in our cohort and their general applicability in CS. PPVs were calculated at cut-off values indicating either the maximal sensitivity or specificity.

In addition to the perioperative outcomes, we also analyzed the characteristics of incremental subgroups of high-risk patients defined by the Risk-E score. With the use of the 36-Item Short Form Survey (SF-36), we evaluated different quality of life (QoL) parameters at latest follow-up for patients with higher estimates of mortality by the Risk-E score (> 45%). This particular score was selected as it was the one that performed better in our cohort.

### Statistical analysis

Variables are expressed as the median and interquartile range [IQR], or as proportions, as appropriate. In-hospital mortality and survival rates were assessed at 30 days, 6 months, and 1 year. Discrimination of the risk scores was studied by performing the receiver operating characteristics (ROC) curve for each risk score and its corresponding area under the curve (AUC). The selected thresholds for each score assessment using ROC curves were obtained maximizing the sum of sensibility and specificity. Calibration for each score was assessed using the Hosmer–Lemeshow goodness-of-fit test. The statistical analysis was performed using STATA statistical software version 14.1 (Stata Corp., College Station, TX).

## Results

A total of 235 patients were evaluated at our institution with acute left-sided infective endocarditis. Data related to our patients who did not undergo CS has been published elsewhere [8]. A total of 142 patients (60%) underwent surgery and 2 patients (1.4%) were lost to follow-up.

### Characteristics of the operated IE cases in the cohort (Table 1)

**Table 1:** Baseline characteristics, microbiology, and perioperative and postoperative variables of 142 patients undergoing cardiovascular surgery for infective endocarditis from 2014 to 2019

	*N*	%
Baseline characteristics		
Age, median (IQR)	64 (30–82)	
Male sex	109	76.8
Diabetes	33	23.2
Hypertension	92	64.8
Stroke related to IE	20	14.1
Previous IE	9	6.3
Symptoms shorter than 1 month	81	57.0
Previous CABG	10	7.0
NYHA III–IV preoperative	95	66.9
Cardiogenic shock	33	23.2
Severe left ventricle dysfunction (LVEF < 30%)	8	5.6
Severe pulmonary hypertension (sPAP > 55 mmHg)	38	26.8
IE Type		
Native	95	66.9
Prosthetic	47	33.1
CA-IE	110	77.5
N-IE	32	22.5
Surgery		
Emergent	38	26.8
Urgent	90	63.4
Aortic involvement	66	46.5
Mitral involvement	35	24.6
Mitro-aortic involvement	41	28.9
Paravalvular complication: abscess	38	26.8
Intracavitary fistula	8	5.6
Valvular perforation	54	38.0
CABG	15	10.6
IVF reconstruction	20	14.1
Other concomitant procedures beyond valvular surgery	14	9.9
Microbiology		
Viridans group streptococci	41	28.9
Coagulase-negative staphylococci	30	21.1
* Staphylococcus aureus*	19	13.4
Enterococci	21	14.8
* Streptococcus gallolyticus*	10	7.0
Other	18	12.7
Negative culture	3	2.1
Postoperative complications		
Mechanical ventilation > 48 h	28	19.7
Perioperative myocardial infarction	7	4.9
Return to theatre (bleeding)	9	6.3
Permanent pacemaker implant	12	8.5
Stroke		
Ischemic	2	1.4
Hemorrhagic	7	4.9
Renal failure requiring RRT	19	13.4
Deep wound infection	0	0
Outcome		
In-hospital mortality	8	5.6
Valvular reoperation at follow-up	2	1.4
Reinfection (different microorganisms)	3	2.1
Relapse	0	0
6-month mortality	9	6.3
1-year mortality	13	9.2

*CABG*, coronary artery bypass grafting; *NYHA*, New York Heart Association functional classification; *LVEF*, left ventricle ejection fraction; *sPAP*, systolic pulmonary artery pressure; *CA-IE*, community-acquired infective endocarditis; *N-IE*, nosocomial-acquired infective endocarditis; *IVF*, intervalvular fibrosa; *RRT*, renal replacement therapy

Male gender predominated (76.8%) and median age was 64 years (IQR 30–82). There were 95 cases of native valve IE (66.9%) and 47 cases of prosthetic IE (33.1%). Perivalvular abscess, fistulas, or perforation occurred in 100 cases (70.4%). Twenty cases (14.1%) suffered documented preoperative emboli in the central nervous system and 33 cases (26.2%) presented with cardiogenic shock.

The causative microorganism was identified in 97.9% of patients. The most prevalent causative agent were viridans group streptococci (VGS), followed by coagulase-negative staphylococci (CoNS) and *Staphylococcus aureus*. These results show a tendency in higher number of CoNS and a lower frequency of *S. aureus* as causative microorganisms, which are in line with the most recent literature [29, 30].

### Postoperative outcomes

In-hospital and 30-day mortality was 5.6% (eight patients). The mortality reasons and deceased patients’ profile are reported in Supplementary Table 1. Six-months and 1-year survivals were 93.7% and 90.8%, respectively. No relapses of the index infection were observed at follow-up. Median follow-up was 33.1 months (IQR 18.6–49.9).

### Risk score data and performance in our cohort of patients

**Fig. 1:**
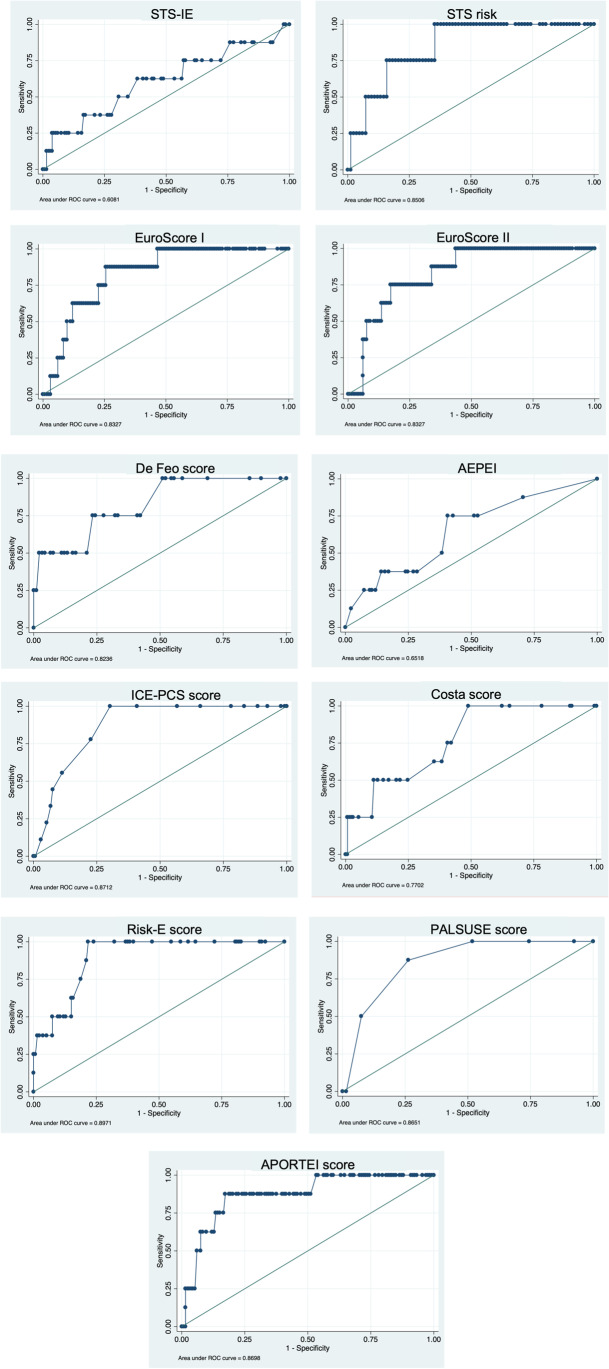
ROC curves and AUC for the scores analyzed. The risk score with the best performance was Risk-E score (AUC = 0.89), ICE-PCS score (AUC = 0.87), APORTEI score (AUC = 0.87), and PALSUSE score (AUC = 0.87). The worst performance was found on the STS-IE risk score (AUC = 0.61)

The mean, range, standard deviation, and CI for each risk score model are summarized in Table 2. The ROC curve and its AUC for the risk scores are shown in Fig. 1. The majority of IE scores showed a high estimated risk of mortality in our cohort, between 15 and 30%. The risk scores with lower estimated mortality were STS (5.74–10.18%) and De Feo-Cotrufo score (9.2–27.3%). Meanwhile, the risk scores with higher predicted mortality were EuroScore I, Costa, and APORTEI. The risk score with the best performance was Risk-E (AUC = 0.89, 95% CI 0.823–0.970) and the one with worst performance was the STS-IE risk score (AUC = 0.61, 95% CI 0.370–0.846). When tested in our cohort, all scores appeared to show adequate calibration (Supplementary Table 2). The number of operated patients whose risk exceeded arbitrary cut-off points for every score is summarized in Table 3. Survival for the highest cut-off values remains ≥ 79%. Similarly, the PPV and the 95% CI of the scores studied are shown in Table 4. As the risk score increased, the PPV also increased, indicating better discrimination. However, the PPVs calculated were all consistently low between scores.

**Table 2:** Performance of risk scores for in-hospital mortality and composite outcomes after surgery for infective endocarditis

Risk Score	Mean score value (95% CI)	SD	Range	Expected mortality % (range)	Observed mortality % (95%CI)	AUC	95% CI
EuroScore I [25]	35.2 (31–39.4)	25.38	2.38–89.7	35.2	**5.6 (1.8**–**9.4)**	0.83	0.72–0.94
EuroScore II [24]	23.5 (19.9–27)	21.45	1.27–85	23.5	**5.6 (1.8**–**9.4)**	0.83	0.72–0.94
STS-risk^a^ [26, 27]	7.9 (5.7–10.2)	10.38	0.28–74.7	7.96	5.0 (0.7–9.2)	0.85	0.69–1.00
STS-IE risk^b^ [28]	38.5 (5.7–10.1)	15.73	10–91	10–20*	**5.6 (1.8**–**9.4)**	0.61	0.37–0.85
Risk-E [11]	21.6 (19.5–28.9)	7.60	0–62	24.1	**5.6 (1.8**–**9.4)**	0.89	0.82–0.97
PALSUSE [5]	2.6 (2.4–2.9)	1.45	0–6	16–30*	**5.6 (1.8**–**9.4)**	0.87	0.77–0.96
APORTEI [12]	45.7 (41.7–49.7)	24.5	0–102.5	20 (10–40)*	**5.6 (1.8**–**9.4)**	0.87	0.75–0.99
De Feo-Cotrufo^c^ [10]	17.1 (15.1–19.1)	9.9	0–46	9.2–27.3*	**4.2 (0.2**–**8.3)**	0.82	0.61–1.00
Costa [9]	15.1 (13.8–16.3)	7.7	0–38	32.7–56.5*	**5.6 (2.8**–**10.7)**	0.77	0.63–0.91
AEPEI [13]	1.9 (1.7–2.3)	1.9	0–6.6	9–18.90*	5.6 (2.8–10.7)	0.65	0.45–0.86
ICE-PCS^d^ [14]	9.2 (8.6–9.8)	3.7	0–18	30*	**6.3 (2.3**–**10.4)**	0.87	0.79–0.95
STS-IE composite risk [28]	35.5 (32.9–38.1)	15.7	7–83	60–70*	30.3 (22.7–37.8)	0.67	0.58–0.76

Results shown in bold reflect statistically significant differences between expected and observed mortality

^a^The STS-risk score only is applied in patients without multivalvular involvement

^b^STS-IE composite risk includes the risk of mortality and major complications after surgery

^c^The De Feo-Cotrufo score is applied only in patients with native valve IE

^d^The ICE-PCS score is evaluated at 6-month mortality

^*^Expected mortality is given as % and/or as a range according score prediction

**Table 3:** Cross-validation of risk scores with hypothetical cut-off values and outcomes of operative management

	Arbitrary cut-off points					
	> 45%		> 60%		> 70%	
	*Patients operated*	*Survival*	*Patients operated*	*Survival*	*Patients operated*	*Survival*
EuroScore I [25]	42	83%	33	85%	19	79%
EuroScore II [24]	24	79%	13	77%	6	100%
Risk-E [11]	27	81%	21	81%	14	79%
PALSUSE* [5]	77	91%	NA	-	NA	-
APORTEI* [12]	19	74%	NA	-	NA	-
De Feo-Cotrufo [10]	25	92%	14	85%	13	84%
Costa [9]	NA	-	60	92%	33	88%
AEPEI [13]	10	90%	4	75%	NA	-
ICE-PCS [14]	38	81%	12	75%	5	80%

The STS score is excluded due to only one patient exceeding 45% of mortality risk. The risk estimated for this patient was 74.7% and was a survivor. STE-IE was also excluded due to low performance. *APORTEI and PALSUSE scores only estimated risk above of 45%

**Table 4:** Positive predictive values and 95% CI of EuroScore II, STS-risk score, Risk-E score, and ICE-PCS score. Score’s cut-off values were defined according to the maximum possible specificity or sensitivity

	Sensitivity	Specificity	Mortality (true positive)	False positive	Predictive positive value	95% CI
EuroScore II (cut-off)						
> 18.47	100	56.39	8	58	12.12	4.1–20.2
> 58	50	92.48	4	10	28.57	4.4–52.7
STS-risk IE (cut-off)						
> 5.84	100	64.4	4	29	12.1	0.8–23.5
> 18.56	50	93.9	2	5	28.6	0.0–62.7
Risk-E (cut-off)						
> 28	100	78.2	8	29	21.62	8.1–35.2
> 47	37.5	98.5	3	2	60	16.2–100
ICE-PCS (cut-off)						
> 11	100	69.7	9	40	18.4	7.3–29.4
> 14	44.4	92.4	4	10	28.6	4.4–52.7

### Profile, 1-year survival, and QoL of high-risk patients according to the Risk-E score

We found increasing proportions of *S. aureus* etiology, cardiogenic shock, thrombocytopenia, and acute renal failure as the predicted risk raised. Patient’s characteristics for each group at incremental risks are detailed in Supplementary Table 3. In-hospital mortality slightly increased as the risk rose but remained low compared to estimated mortality. In the highest risk group (> 70%), only one patient died in the first year after surgery. Further analysis of QoL subcomponents for patients with Risk-E > 45 is shown in Supplementary Table 4. Follow-up data was obtained at a median of 37.0 months after surgery [IQR = 19.0–52.7]). On a global evaluation, this subgroup of patients performed slightly lower than the general population in the physical subcomponent evaluation and similarly in the mental subcomponent [31].

## Discussion

We externally assessed the discriminative power of the currently available IE risk scores in a center with an Endocarditis Team active for more than 30 years. From a statistical point of view, the general discrimination of most risk scores in our cohort appears appropriate. However, the difference between predicted and observed mortalities struck from a clinical and statistical standpoint. The Endocarditis Team through an experienced, collegiate, and multidisciplinary care may account for such outcomes. Prior data from the group of Botelho-Nevers et al. showed a significative reduction in 1-year mortality (18.5 to 8.2%, *p* = 0.008) after implementing a management-based approach through standardized diagnostic and therapeutic protocol on patients with diagnosis of IE [32]. Therefore, we hypothesize that the presence of an IE Team as well as undergoing timely cardiac surgery might be used as a correction factor for predicting mortality in further scoring systems.

We acknowledge that none of the available scores has influenced our IE practice. Conceptually, the ability to count on a perfect score when complex IE decision-making is required would prevent denial of surgery to a viable patient and limit unnecessary escalation. In our experience, mortality after surgery for endocarditis arises from brain bleeding from preoperative embolism, severely depressed systolic function not amenable to transplantation/mechanical circulatory support, poor tissue-suture anchorage, and advanced patient directives. Important features impacting survival after cardiac surgery (e.g., non-reversible pulmonary hypertension, overestimated ventricular function with multiple valve regurgitation, calcified aorta, need for extended cardiac ischemic times, and coronary disease not amenable to revascularization) have been ignored from scoring systems [33–35].

Understanding how risk scores have been created provides clues on the current study findings. Most of the scores include patients with right- and left-sided IE (De Feo score, PALSUSE score) and different stages of the disease and merge patients with healed IE and patients undergoing medical treatment alone (De Feo score, PALSUSE score, AEPEI score, Costa score, ICE-PCS), or general cardiac patients (STS score, EuroScore I and II). Our cohort’s risk profile is higher than those that served to formulate the majority of scores and most of the surgically reported experiences [7, 36]. The reported mortality rates vary from 8.2% (STS-IE) [13] to 28.3% (RISK-E) [9], being De Feo-Cotrufo and STS-IE the only ones with mortality below 10% [10, 28]. Wang and colleagues [(18)] concluded that EuroScore I overestimated the risk in IE and failed to discriminate operative mortality. Varela et al. added that EuroScore II underestimated mortality in patients with low risk most likely as a result of not capturing important inherent features of IE patients [2]. Other studies showed similar results in which EuroScore II tended to underestimate mortality by 5–10% when predicted mortality was greater than 10% for IE patients [37]. A recent meta-model with weighted IE-specific variables from individual scores from Fernández-Félix et al. showed an increased discriminatory power compared to their previous existent scores [38].

The score proposed by De Feo-Cotrufo [10] is the only score included in current clinical guidelines [7]. It arises from patients operated over a 30-year period and is limited to native valve IE. Wang also demonstrated its applicability could be extended to prosthetic valve IE patients [18]. The Costa score [9] has been found to have poor performance in our cohort. We believe that the different patient’s characteristics of both cohorts (Brazilian population, mean age of 33.9 years; and patients in whom surgery was not performed) may be reasons for the observed results. The ICE-PCS score is the only one that predicts mortality at 6 months [14]. Similarly, to other scores, it merges patients undergoing surgery (48.1%) with patients treated only medically. For patients not undergoing surgery, this score has demonstrated reasonable prediction of mortality [16]. The PALSUSE [5], RISK-E [11], and APORTEI [12] scores have the best performance in our cohort. Similar preoperative characteristics and inclusion of IE-specific and critical preoperative variables may explain our findings.

Contemporary IE clinical guidelines state clearly the theoretical indications to pursue surgery [7]. However, it is acknowledged that in practice the final decision to offer surgery usually relies on the patients’ condition and risk profile. We previously reported our non-operated cohort and compared it with operated patients [8]. In our experience, the estimated risk profile of patients not undergoing surgery (despite contemporary guidelines indication) was significantly lower than of those operated (EuroScore II 9.4% vs 23.3%, *p* < 0.007). Over a similar time period, a total of 46 patients with acute left-sided IE with indications of surgery did not undergo surgery, which accounted for the 27% of patients with formal indication of surgery dictated by guidelines. We also reviewed the reasons for not pursuing surgery and those usually overlapped, but a high score was never a motivation itself. Thirty-day, 1-year, and 2-year mortality were 63%, 85%, and 90%, respectively. Recent data suggest that the implementation of several risk scores, including EuroScore II, might be useful in predicting mortality even in not operated patients [39]. In our experience, EuroScore II seems to underestimate mortality in non-operated patients while overestimates morality in surgical patients. Importantly, the reasons for not undergoing surgery in this cohort of non-operated patients usually include cerebral brain bleeding, end-stage cancer, unwillingness to undergo surgery, and end-stage liver disease, among other reasons that are not usually captured by prediction scores.

If a high numeric risk score was to be used as a sole tool to deny surgery, we attested that preventable deaths might have occurred. The perfect score would be a tool with 100% specificity in death prediction so that surgery is not even undertaken. Anything below this capability will lead to questioning how much risk is reasonable to accept. Undeniably, at times, the borders of operability and futility remain arbitrary. As for any urgent cardiac intervention, the likelihood of survival and return to previous status relies on the extent of biological reserve and organ dysfunction acuity. We evaluated not only mortality but also QoL in this very high-risk subgroup to understand whether futile interventions were pursued. In those high-risk patients, data on survival beyond the acute phase along with follow-up QoL points at a beneficial and appropriate use of surgery.

Procedural reporting of mortality has been associated with surgical risk-averse behavior as it poses a challenge to the surgeon at an individual level. At a departmental and institutional level, public reporting of outcomes and benchmarking through inaccurate risk scores threaten the best interest of patients. A charitable decision erring on the side of the patient is the logical course of action regardless of high estimates of death, if eloquent recovery with surgery is possible. The need for improved scores remains to guide resource allocation and the referral of surgical candidates to the best-performing IE teams.

### Limitations

The first is the single-center observational retrospective nature of the study, although the local Endocarditis Team prospectively evaluated all patients. Second, given that our institution acts as a referral center for endocarditis, the pattern of surgical candidates may be biased towards patients with different stages of the disease and more complex interventions that were at times denied elsewhere. However, it is possible that this occurred also to centers participating in the production of risk scores. The low number of adverse outcomes challenges the analysis of the different risk scores. Finally, the QoL assessment has occurred at variable time frames since CS was pursued. Thus, if QoL assessment is obtained years after the operation, unmodifiable natural events—such as aging itself or progressive organ dysfunction—not related to the CS-IE may have led to worse punctuation.

## Conclusion

The observed mortality in our cohort is significantly lower than predicted by contemporary risk scores. Despite the reasonable numeric performance of the analyzed scores, their utility in judging the operability of a given patient remains questionable, as is demonstrated in the cross-validation analysis. On the sole basis of a high-risk value, many patients would be denied a lifesaving operation upholding the potential to restore QoL. Individual assessment of risk from a specialized team might improve outcomes in this complex subset of patients. Future IE guidelines may recommend that denial of surgery should only follow a highly experienced Endocarditis Team evaluation.

## Supplementary information

Below is the link to the electronic supplementary material.

Supplementary file1 (DOCX 44 KB)

## Data Availability

The datasets generated and/or analyzed during the current study are not publicly available due to potential individual privacy disruption but are available from the corresponding author on reasonable request.

## Code availability

The authors declare that the data supporting the findings of this study are available within the article and its supplementary information files.
