# Supplementary material for: Risk scores’ performance and their impact on operative decision-making in left-sided endocarditis: a cohort study
Source: Eur J Clin Microbiol Infect Dis. 2022 Nov 8;42(1):33–42. doi: 10.1007/s10096-022-04516-2 (PMC9816251; doi:10.1007/s10096-022-04516-2)
Supplement: Supplementary file 1 — Supplementary file1 (DOCX 44 KB) [file 10096_2022_4516_MOESM1_ESM.docx]

**Supplementary Table 1**: Clinical characteristics and cause of death of deceased patients

| Patient | Age | LVEF | sPAP | | EuroScore-II | Microorganism | Involvement | Surgery | | Cause of death |
| --- | --- | --- | --- | --- | --- | --- | --- | --- | --- | --- |
| 1 | 71 | 65 | 70 | 66.8 | | *S. aureus* | PVE Mi | Mi valve replacement + T valve repair + lead extraction | | Refractory heart failure (severe preoperative right ventricle dysfunction) not amenable to heart transplantation |
| 2 | 74 | 55 | 45 | 24.1 | | *S. aureus* | Invasive Mi | Mi valve replacement | | Preoperative brain embolic event and postoperative hemorrhagic stroke |
| 3 | 72 | 35 | 33 | 46.3 | | *S. epidermidis* | PVE Ao + Peri annular abscess | Ao valve replacement | | Multiple major comorbidities. Advanced directives, withdrawal of care at family request when facing need for dialysis. |
| 4 | 68 | 55 | 33 | 18.5 | | *S. aureus* | Invasive Mi + annular abscess | Mi valve replacement + annular reconstruction with bovine pericardium | | Preoperative posterior atrioventricular groove disruption and hemopericardium due to invasive mitral endocarditis. Died of recurrent disruption with hemodynamic compromise not amenable to heart transplantation |
| 5 | 74 | 55 | 46 | 64.4 | | *S. epidermidis* | PVE Ao + Mi + T + IVF abscess | Aortic root replacement with homograft + Mi valve replacement + T valve repair + IVF reconstruction + CABG + pacemaker extraction | | Extensive comorbidities and poor preoperative left ventricular function. Postoperative septic shock (catheter sepsis) |
| 6 | 79 | 45 | 41 | 58.0 | | *E. faecalis* | PVE Ao + Fistula + IVF abscess | Aortic root replacement with homograft + IVF reconstruction + Mi and T valve repair + CABG | | Sudden cardiac tamponade due to delayed (1 week after surgery) aortoventricular disruption. |
| 7 | 76 | 55 | 31 | 41.9 | | *S. epidermidis* | Ao + annular abscess | Aortic valve replacement + Mi valve repair + abscess debridement | | Preoperative dialysis. Hemorrhagic stroke and withdrawal of care at family request. |
| 8 | 56 | 55 | 60 | 66.1 | | *S. mitis* | Ao + T + Fistula + Abscess periaortic | | Aortic root replacement + T valve replacement + fibrous body reconstruction with bovine pericardium + CABG | Preoperative aorto-right atrium fistula. Refractory heart failure with right ventricle dysfunction under right ventricle assist device not candidate for heart transplant |

Abbreviations: LVEF, Left Ventricular Ejection Fraction; sPAP, systolic Pulmonary Artery Pressure; PVE, Prosthetic Valve Endocarditis; Ao, Aortic valve; Mi, Mitral valve; T, Tricuspid valve; IVF, Intervalvular Fibrosa; CABG, Coronary Artery Bypass Grafting.

**Supplementary Table 2**: Performance of the predictive scores by the Hosmer-Lemeshow test.

| Risk Score | Chi-square | Degrees of freedom | *p* value |
| --- | --- | --- | --- |
| EuroScore I (25) | 3.33 | 10 | 0.91 |
| EuroScore II (24) | 2.87 | 10 | 0.94 |
| STS risk (26,27) | 4.59 | 10 | 0.80 |
| STS-IE risk (28) | 5.15 | 10 | 0.74 |
| Risk-E (11) | 9.54 | 10 | 0.30 |
| PALSUSE (5) | 0.47 | 5 | 0.93 |
| APORTEI (12) | 5.23 | 10 | 0.73 |
| De Feo-Cotrufo (10) | 5.08 | 10 | 0.75 |
| Costa (9) | 12.49 | 9 | 0.09 |
| AEPEI (13) | 8.34 | 8 | 0.21 |
| ICE-PCS (14) | 4.67 | 10 | 0.79 |
| STS-IE composite risk (28) | 11.64 | 10 | 0.17 |

**Supplementary Table 3:** Baseline characteristics, microbiology, perioperative and postoperative variables of high-risk patients categorized by Risk-E score.

|  | Mortality risk > 45%  N = 27 | Mortality risk > 60%  N = 21 | | Mortality risk > 70%  N = 14 | |
| --- | --- | --- | --- | --- | --- |
| BASELINE CHARACTERISTICS |  |  | |  | |
| Age, median (IQR)^§^ | 67  (61-71.5) | | 64  (60-69) | | 63  (58.5-68.8) |
| Male gender | 19 (70.4) | | 14 (66.7) | | 7 (50) |
| Diabetes | 7 (25.9) | | 6 (28.6) | | 4 (28.6) |
| Hypertension | 23 (85.2) | | 17 (81.0) | | 12 (85.7) |
| Embolism | 9 (33.3) | | 7 (33.3) | | 4 (28.6) |
| Acute renal insufficiency^§^ | 16 (59.6) | | 15 (71.4) | | 11 (78.6) |
| Symptoms (less than one-month) | 19 (70.4) | | 16 (76.2) | | 11 (78.6) |
| Thrombocytopenia^§^ | 11 (40.7) | | 10 (47.6) | | 9 (64.3) |
| Septic shock^§^ | 9 (33.3) | | 8 (38.1) | | 4 (28.6) |
| Cardiogenic shock^§^ | 24 (88.9) | | 20 (95.2) | | 14 (100) |
| Preoperative mechanical ventilation | 14 (51.9) | | 12 (57.1) | | 8 (57.1) |
| IE TYPE |  | |  | |  |
| Prosthetic^§^ | 11 (40.7) | | 9 (42.9) | | 6 (42.9) |
| Native | 7 (25.9) | | 4 (19.0) | | 3 (21.4) |
| SURGERY |  | |  | |  |
| Emergent | 13 (48.1) | | 9 (42.9) | | 4 (28.6) |
| Urgent | 13 (48.1) | | 11 (52.4) | | 9 (64.3) |
| Aortic involvement | 13 (48.1) | | 8 (38.1) | | 3 (21.4) |
| Mitral involvement | 6 (22.2) | | 6 (28.6) | | 5 (35.7) |
| Mitro-aortic involvement | 8 (29.6) | | 7 (33.3) | | 6 (42.9) |
| Paravalvular complication: abscess^§^ | 10 (37.0) | | 6 (28.6) | | 4 (28.6) |
| Paravalvular complication: fistula^§^ | 4 (14.8) | | 2 (9.5) | | 1 (7.1) |
| Other concomitant procedures beyond valvular surgery | 13 (48.1) | | 10 (47.6) | | 7 (50.0) |
| MICROBIOLOGY |  | |  | |  |
| Viridans group Streptococci | 7 (25.9) | | 5 (23.8) | | 1 (7.1) |
| *Staphylococcus aureus*^§^ | 10 (37.0) | | 10 (47.6) | | 8 (57.1) |
| POSTOPERATIVE COMPLICATIONS | | | | | |
| Mechanical ventilation >48 hours | 13 (48.1) | | 11 (52.4) | | 8 (57.1) |
| Perioperative myocardial infarction | 3 (11.1) | | 1 (4.8) | | 0 (0) |
| Return to theatre (bleeding) | 4 (14.8) | | 3 (14.3) | | 3 (21.4) |
| Permanent pacemaker implant | 3 (11.1) | | 2 (9.5) | | 0 (0) |
| Stroke (ischemic or hemorrhagic) | 3 (11.1) | | 2 (9.5) | | 2 (14.3) |
| Ischemic | 1 (3.7) | | 0 (0) | | 0 (0) |
| Hemorrhagic | 2 (7.4) | | 2 (9.5) | | 2 (14.3) |
| Renal failure requiring RRT | 12 (44.4) | | 10 (47.6) | | 8 (57.1) |
| Deep wound infection | 0 (0) | | 0 (0) | | 0 (0) |
| OUTCOME |  | |  | |  |
| In-hospital mortality | 5 (18.5) | | 4 (19.0) | | 3 (21.4) |
| Valvular reoperation at follow-up | 0 (0) | | 0 (0) | | 0 (0) |
| Reinfection (different microorganism) | 1 (3.7) | | 1 (4.8) | | 1 (7.1) |
| Relapse | 0 (0) | | 0 (0) | | 0 (0) |
| NYHA I-II at follow-up | 20 (90.9) | | 14 (87.5) | | 10 (90.9) |
| 6-month mortality | 5 (18.5) | | 4 (19.0) | | 3 (21.4) |
| 1-year mortality | 6 (22.2) | | 5 (23.8) | | 4 (28.6) |

Abbreviations: CABG, Coronary Artery Bypass Grafting; NYHA, New York Heart Association functional classification; CA-IE, Community Acquired Infective Endocarditis; N-IE, Nosocomial acquired Infective Endocarditis; IVF, Intervalvular Fibrosa; RRT, Renal Replacement Therapy.

Variables are defined as Olmos et al. described in their score (11). ^§^ correspond to variables included in Risk-E score.

|  | **Mean** ± **Std. Err.** | **95% Confidence interval** | **Reference values [29]** | **% Very good or more** | **% Good** | **% Fair or less** | **% Ceiling** | **% Floor** |
| --- | --- | --- | --- | --- | --- | --- | --- | --- |
| **Composite scores** |  | | | | | | | |
| PCS | 38.8 ± 2.7 | 44.2 – 33.4 |  |  |  |  |  |  |
| MCS | 48.2 ± 3.1 | 54.4 – 42.1 |  |  |  |  |  |  |
| **Physical data** |  | | | | | | | |
| Physical Functioning | 52.4 ± 7.4 | 67.1 – 37.6 | 66.6 – 64.8 | 26.3 | 47.4 | 26.3 | 5.3 | 5.3 |
| Role-Physical | 50.0 ± 10.6 | 71.3 – 28.7 | 74.6 – 72.0 | 42.1 | 10.5 | 47.4 | 42.1 | 36.8 |
| Bodily Pain | 67.3 ± 6.3 | 79.8 – 54.7 | 69.4 – 67.4 | 36.8 | 57.9 | 5.3 | 31.6 | 5.3 |
| General Health | 53.7 ± 6.3 | 66.4 – 41.0 | 56.6 – 55.2 | 15.8 | 68.4 | 15.8 | 5.3 | 5.3 |
| **Mental data** |  | | | | | | | |
| Social Functioning | 64.5 ± 8.0 | 80.4 – 48.6 | 80.1 – 78.3 | 42.1 | 36.8 | 21.1 | 42.1 | 5.3 |
| Role-Emotional | 70.2 ± 9.8 | 89.8 – 50.5 | 85.4 – 83.8 | 63.2 | 15.8 | 21.1 | 63.2 | 21.1 |
| Vitality | 58.2 ± 6.4 | 70.9 – 45.4 | 61.3 – 59.7 | 26.3 | 57.9 | 15.8 | 5.3 | 5.3 |
| Mental Health | 72.8 ± 5.5 | 83.7 – 61.9 | 69.0 – 67.6 | 42.1 | 47.4 | 10.5 | 2 1.1 | 5.3 |

**Supplementary Table 4:** Quality of life assessed by SF-36 questionnaire at follow-up for patients with Risk-E > 45 (n=19).

Abbreviations: PCS, physical summary component; MCS, mental summary component.
